# Round the Hospitals

**Published:** 1917-03-17

**Authors:** 


### ROUND THE HOSPITALS.

THE Matrons of Poor-Law Infirmaries, Superintendent Nurses, and Sisters of the Poor-Law Service, if they are wise, will lose no time in organising the nurses under their control to defeat the attempt now being made by the National Poor-Law Officers' Association, for its own purposes, to capture Poor-Law nurses. If the Association were to succeed, the taint of the Poor Law would be likely to attach to Poor-Law nurses, and would, if it did, mean disadvantage and injury to a large number of fully trained nurses of the best. Miss Barton, President of the Poor-Law Infirmary Matrons' Association, is anxious to organise. To succeed, anxiety must be expressed by action and co-operation. Already meetings have been held at the Kensington Infirmary and Bethnal Green Military Hospital, where the nurses have been addressed by Miss Rundle, as representing the College of Nursing, who was thus enabled to remove the misapprehensions created by the representatives of the National Poor-Law Officers' Association. Time is of the first importance to all Poor-Law nurses at this juncture, and we should be glad to hear that a rota of meetings throughout the Poor-Law Service in defence of the nurses and their rights had been organised by the Poor-Law Infirmary Matrons' Association, and that the co-operation of Miss Cox-Davies and other able speakers with that of the Secretary of the College had been secured. The present is an opportunity for Poor-Law Infirmary Matrons to prove that their first purpose is, as it always has been, to help Poor-Law nurses to help themselves. The nurses' interest is the first great object of the Matrons' Association despite the attempts made to prejudice Poor-Law nurses by emphatic statements to the contrary. We look to the President of the Council of the Poor-Law Infirmary Matrons' Association to work actively to remove this wrong impression. It is this Association's high privilege to do justice to the Matrons and all its members by protecting, counselling, and helping Poor-Law nurses throughout the Service to defend their rights and maintain their independence, in the face of an attempt to coerce and compel Poor-Law nurses, to their undoing, to join the National Poor-Law Officers' Association, with which, properly and wisely, they should have little or no concern.

IT is interesting to observe that the position of the hospitals in New Zealand has been adversely affected by the depletion of the nursing and medical staffs for military duty. One result of this has been that the North Canterbury Hospital Board, sitting at Christchurch, has directed the attention of the Inspector-General of Hospitals and Charities to the fact that the continual withdrawal of the senior officers must seriously mar the efficiency of the hospitals, and that this aspect should be considered before nurses or others are called up for military service, if the civilian hospitals and kindred institutions are to be maintained in a condition of efficiency.

---

IT is stated to be the general practice in New Zealand hospitals, with the exception of the Wellington Hospital, to employ probationers during their first year in wards either wholly or partly reserved for the treatment of infectious or contagious diseases. The Medical Committee of the Auckland Hospital and Charitable Board, at a meeting in November, expressed its opinion that this is a most indefensible and dangerous practice. The infectious or contagious diseases commonly treated in the wards in question are scarlet fever, diphtheria, typhoid fever, measles, pneumonia, influenza, and septicæmia. In the course of a discussion the Chairman, Mr. M. J. Coyle, said a probationer nurse was not employed in any ward of the Auckland Hospital which was wholly used for infectious complaints. Where she was employed in a general ward that contained such cases she was required to give attention to the infectious cases. In a time of epidemic, when a large number of infectious cases were placed in one ward, probationers were not allowed into that ward. This statement strongly supports the wisdom of the opinion expressed by the Medical Committee, which, we regret to see, appears to have been ignored by the Board. A resolution was passed, however, at the instance of the Medical Committee providing for the inoculation of the nurses in general against typhoid fever, making inoculation compulsory and a condition precedent to appointment. The existing rules provide that in New Zealand hospitals vaccination against small-pox is now compulsory for nurses.

### ROUND THE HOSPITALS (*continued*).

These facts are interesting, and illustrate some special difficulties which hospital authorities in the Dominions Beyond Seas have to overcome. This knowledge may make those in the Homeland well content with the efficiency of the administrative system in British hospitals in regard to these matters.

---

THE twenty-sixth annual report of the Nurses' Co-operation has special importance for all nurses and those interested in them at the present time, for it testifies to the extraordinary success which has attended this, the first, effort on behalf of British nurses to co-operate for their own interests and protection. When this Co-operation was first contemplated it was strongly opposed by certain leaders of the nursing profession at the time, who regarded it as being calculated to interfere with the private nursing staffs of certain large hospitals and to be otherwise undesirable. So strong was this feeling that the writer, as one of its founders, was written to by an influential matron in those days, who roundly stated that if he persisted in organising the Nurses' Co-operation she would never speak to him again. Everybody is liable to mistakes, and the most excellent lady in question would, if she were alive and could now study the latest report of the Nurses' Co-operation, probably alter her opinion. There are at present 450 fully trained nurses on the general staff, thirty-five asylum-trained nurses for mental patients, and twenty-two nurses eligible for election working on probation for six months. The total number of cases nursed in 1916 was 5,973, and the total fees received by the nurses during the twelve months was £50,629.

---

THIS is a remarkable result, and the figures may be taken with those we recently published which illustrated the growth of the Royal National Pension Fund for Nurses and recorded the fact that its invested funds amount to £2,070,000, its pension premium income for 1916 to £108,000, the annuities paid in that year being £60,000, in addition to surrender values for policies surrendered by nurses in the same year, which amounted to £63,000. Since it was established the Pension Fund has paid £770,000 in surrender values, a sum very largely in excess of the premiums received from the surrendered policies. There are some 10,000 nurse-members of the Pension Fund.

---

THE Nurses' Co-operation is based on the principle that the nurses on its staff receive the whole of their earnings, subject to a deduction of 5 per cent. in the case of those who joined before 1908 and of 7½ per cent. in the case of those admitted to the staff since that date. The percentage in question is used in the upkeep of the central office, payment of the permanent staff, the maintenance of a policy by which each nurse is insured against the employer's liability under the Workmen's Compensation Act, and other needful expenses. The Co-operation nurses have for eight years derived great benefit from a policy of insurance against sickness and disease, under which each nurse is entitled to a weekly allowance whilst temporarily incapacitated for work. We might bring out other points, but we have said enough to show in a practical way the substantial value of Co-operations for nurses, under good business management. Facts like those quoted should encourage every well-trained nurse of intelligence and independent spirit to register with the Royal British College of Nursing quickly, for recent developments and the continuous progress which they foreshadow may be taken as evidence that when the Royal British College of Nursing is completely organised, it will prove in its working to be the friend, defender, and sure defence of every work-ill-health, or who, from no fault of their own, have confidence in it.

